# Relevance of presenting risks of frailty, sarcopaenia and osteopaenia to outcomes from aneurysmal subarachnoid haemorrhage

**DOI:** 10.1186/s12877-022-03005-7

**Published:** 2022-04-16

**Authors:** Jia Xu Lim, Yuan Guang Lim, Aravin Kumar, Tien Meng Cheong, Julian Xinguang Han, Min Wei Chen, David Wen, Winston Lim, Ivan Hua Bak Ng, Vincent Yew Poh Ng, Ramez Wadie Kirollos, Nicole Chwee Har Keong

**Affiliations:** 1 Department of Neurosurgery, National Neuroscience Institute, Singapore, Singapore; 2 Department of Diagnostic Radiology, Singapore General Hospital, Outram Road, Singapore, 169608 Singapore; 3 Duke-NUS Medical School, Singapore, Singapore

**Keywords:** Subarachnoid haemorrhage, Aneurysm, Outcomes, Frailty, Temporalis muscle thickness, Modified Rankin scale, Zygoma thickness, Intracranial aneurysms

## Abstract

**Introduction:**

Aneurysmal subarachnoid haemorrhage (aSAH) is a condition with significant morbidity and mortality. Traditional markers of aSAH have established their utility in the prediction of aSAH outcomes while frailty markers have been validated in other surgical specialties. We aimed to compare the predictive value of frailty indices and markers of sarcopaenia and osteopaenia, against the traditional markers for aSAH outcomes.

**Methods:**

An observational study in a tertiary neurosurgical unit on 51 consecutive patients with ruptured aSAH was performed. The best performing marker in predicting the modified Rankin scale (mRS) on discharge was selected and an appropriate threshold for the definition of frail and non-frail was derived. We compared various frailty indices (modified frailty index 11, and 5, and the National Surgical Quality Improvement Program score [NSQIP]) and markers of sarcopaenia and osteopaenia (temporalis [TMT] and zygoma thickness), against traditional markers (age, World Federation of Neurological Surgery and modified Fisher scale [MFS]) for aSAH outcomes. Univariable and multivariable analysis was then performed for various inpatient and long-term outcomes.

**Results:**

TMT was the best performing marker in our cohort with an AUC of 0.82, Somers’ D statistic of 0.63 and Tau statistic 0.25. Of the frailty scores, the NSQIP performed the best (AUC 0.69), at levels comparable to traditional markers of aSAH, such as MFS (AUC 0.68). The threshold of 5.5 mm in TMT thickness was found to have a specificity of 0.93, sensitivity of 0.51, positive predictive value of 0.95 and negative predictive value of 0.42. After multivariate analysis, patients with TMT ≥ 5.5 mm (defined as non-frail), were less likely to experience delayed cerebral ischaemia (OR 0.11 [0.01 – 0.93], *p* = 0.042), any complications (OR 0.20 [0.06 – 0.069], *p* = 0.011), and had a larger proportion of favourable mRS on discharge (95.0% vs. 58.1%, *p* = 0.024) and at 3-months (95.0% vs. 64.5%, *p* = 0.048). However, the gap between unfavourable and favourable mRS was insignificant at the comparison of 1-year outcomes.

**Conclusion:**

TMT, as a marker of sarcopaenia, correlated well with the presenting status, and outcomes of aSAH. Frailty, as defined by NSQIP, performed at levels equivalent to aSAH scores of clinical relevance, suggesting that, in patients presenting with acute brain injury, both non-neurological and neurological factors were complementary in the determination of eventual clinical outcomes. Further validation of these markers, in addition to exploration of other relevant frailty indices, may help to better prognosticate aSAH outcomes and allow for a precision medicine approach to decision making and optimization of best outcomes.

**Supplementary Information:**

The online version contains supplementary material available at 10.1186/s12877-022-03005-7.

## Background

Aneurysmal subarachnoid haemorrhage (aSAH) is a condition with significant morbidity and mortality [1], and is associated with substantial economic and disease burden [2]. The decision-making process behind the management of aSAH is complex with outcomes being influenced by the interaction between disease, patient, and doctor factors. It is a process of managing not only the primary brain injury caused during the rupture of the aneurysm, but also balancing the risk of intervention versus rerupture, as well as monitoring for and treatment of the complications of the rupture, including delayed cerebral ischemia. There is a need to triage patients appropriately to avoid over- or under-treating patient cohorts. Age, and grading scales such as the World Federation of Neurosurgical Societies (WFNS) [3] and modified Fisher scale [4], have been shown to be good prognosticators of outcomes and mortality in this condition, and proven utility in guiding treatment intent.

Across the spectrum of surgical interventions, frailty is a concept gaining momentum within the medical community due to the emerging evidence regarding the relevance of frailty risks towards both perioperative and longer-term adverse outcomes [5–8]. Defined as a “biologic syndrome of decreased reserve and resistance to stressors, frailty is thought to result from cumulative declines across multiple physiological systems [9].” Multiple frailty scores exist, with various forms of clinical and instrumental assessment of each of the domains involved, contextualised in various forms of specialties [10]. The modified frailty index – 11 [11] (MFI-11), MFI-5 [12], and the National Surgical Quality Improvement Program (NSQIP) score [13], are three scores derived from the American College of Surgeons NSQIP database (https://www.facs.org/quality-programs/acs-nsqip), that were shown to have strong predictive ability amongst various surgical specialties. Other indices include the clinical frailty score [14], risk analysis index [15], hospital frailty risk score [16], and the FRAIL questionnaire [17], which predicts survival and complications for elective surgery; 30-day mortality and readmission, and duration of hospital stay in the elderly; functional outcome and mortality in elderly trauma patients, respectively. In neurosurgery, the concept of frailty has been explored in various subspecialty conditions including brain tumour [18, 19], chronic subdural haematoma [20], and spine surgery [21–23]. Although some work has been published on aSAH in the frail [24, 25] and in the elderly [26, 27], there has been no study comparing the various indices on the outcomes of this condition.

Sarcopaenia and osteopaenia, the loss of muscle and bone mass, has been noted to be associated with frailty [28, 29]. Temporalis thickness (TMT), a surrogate marker of sarcopaenia, have been shown to correlate with outcomes of various forms of cranial neurosurgery, including glioblastoma [30–32] and various types of brain metastasis [33–35]. TMT has also been noted to be associated with the presentation and functional outcome on discharge in aSAH [36, 37]. Zygoma thickness (ZGM), a proxy for osteopaenia, on the other hand, has been found to correlate with stay in hospital and intensive care as well as ventilator use in patients with mandibular fracture [38].

In this study, we aimed to investigate subarachnoid haemorrhage due to ruptured intracranial aneurysm and the effects of established frailty indices (MFI-11, MFI-5, and NSQIP score) and markers of sarcopaenia and osteopaenia (TMT and ZGM), and compare them against traditional markers of aSAH severity (age, WFNS and Fisher grade). This has implications on the patient risk stratification when considering interventions for aSAH.

## Methods

### Study design and patient selection

This was an observational study conducted by retrospective review of the electronic medical records of patients with aSAH in a tertiary neurosurgical unit between January 2014 to December 2015. Institutional review board review for consent waiver was obtained and the study was performed in accordance with the Declaration of Helsinki. All adult patients with ruptured intracranial aneurysms managed in our centre during this period were included. We excluded patients managed with palliative intent and those lost to follow up.

### Frailty score derivation

Frailty indices were employed in our study included MFI-11 [10], MFI-5 [11], NSQIP [12]. These indices were selected due to multiple studies demonstrating correlation with peri-operative outcomes across multiple surgical pathologies, independently. In addition, these indices do not require extraordinary investigations such as grip strength or walking speed [37], and hence could be reliably derived from patient’s electronic medical records (see Table 1 (Supp) for Variables involved in selected frailty indices).

### Temporalis and zygoma thickness derivation

The TMT was derived from the presenting computed tomography angiography of the circle of Willis (CTA) of each patient in accordance with the description in previous studies [34, 35]. ZGM, being adapted from on a previous study [36], was measured at the midpoint of the zygomatic bone using the bone window axial image. The methodology for both measurements, including our refinements to derive TMT for this study, have been described in detail in the Supplementary Notes.

### Data collection

The electronic medical records of each patient were accessed and baseline demographic information, including the age, gender, smoking status, premorbid functional status (based on modified Rankin scale [mRS]), medical comorbidities (based on Charlson comorbidity index [CCI]), and clinical variables required for each frailty index was recorded. Other information included presenting Glasgow coma scale (GCS), pupillary reactivity, severity of SAH (based on WFNS and Fisher grading), presence of hydrocephalus, and the type of intervention performed. WFNS grades of 1 – 3 are considered good grade.

The CTA findings of TMT and ZGM were evaluated by two study members (JXL and TMC) and the values were interrogated using Cohen’s kappa for inter- and intra-rater agreement. The detailed description of this process as well as the cut-off derivation of TMT and ZGM are further detailed in the Supplementary Notes.

Inpatient and long-term outcomes were also documented. These included the neuroscience intensive care unit (NICU) and overall length of stay, the presence of complications (any infective complications, intervention or non-intervention related; any delayed cerebral ischaemia; any overall complication that is directly due to intervention performed). The need for tracheostomy and ventriculoperitoneal shunt, discharge location, mortality, and functional status and various timepoints were also noted. Functional outcomes were measured using mRS and a favourable mRS was defined using independent ambulation (mRS 0 – 3).

### Statistical analysis

Receiver operating characteristic (ROC) analysis was performed for each marker versus the rate of favourable mRS on discharge, and model performance indices including area under the ROC curve (AUC), Somer’s D statistic and Tau statistic were reported. An appropriate threshold for the best performing marker was subsequently derived using Youden’s index to optimise the sensitivity, specificity, positive and negative predictivce values. The cohort was then divided into frail and non-frail based on this. In addition, the agreement amongst each marker, using Spearman’s correlation, was assessed.

Categorical variables were described using frequency and percent; and continuous variables were reported as mean and standard deviation (or median with 1^st^ and 3^rd^ quartiles, where appropriate). Baseline characteristics were compared between the frail and non-frail groups using Chi square test (or Fisher exact test, where appropriate) and two-sample t-test (or Mann–Whitney U test, depending on whether normality assumption was tenable) for categorical and continuous variables, respectively. Univariate logistic regression analysis was performed to investigate the association with baseline characteristics and clinical features. Multivariable logistic regression was then conducted to adjust for potential confounders identified from the literature or univariate analysis. In view of the small sample size, only WFNS grade was included in the adjustment for multivariable analysis. Un-adjusted and adjusted odds ratios (OR) and 95% confidence intervals (CI) were reported. Data analysis was performed using SAS software version 9.4 for Windows (Cary, NC: SAS Institute INC.) and statistical significance was set at *p* < 0.05.

## Results

### Marker selection and the definition of frailty

**Fig. 1:**
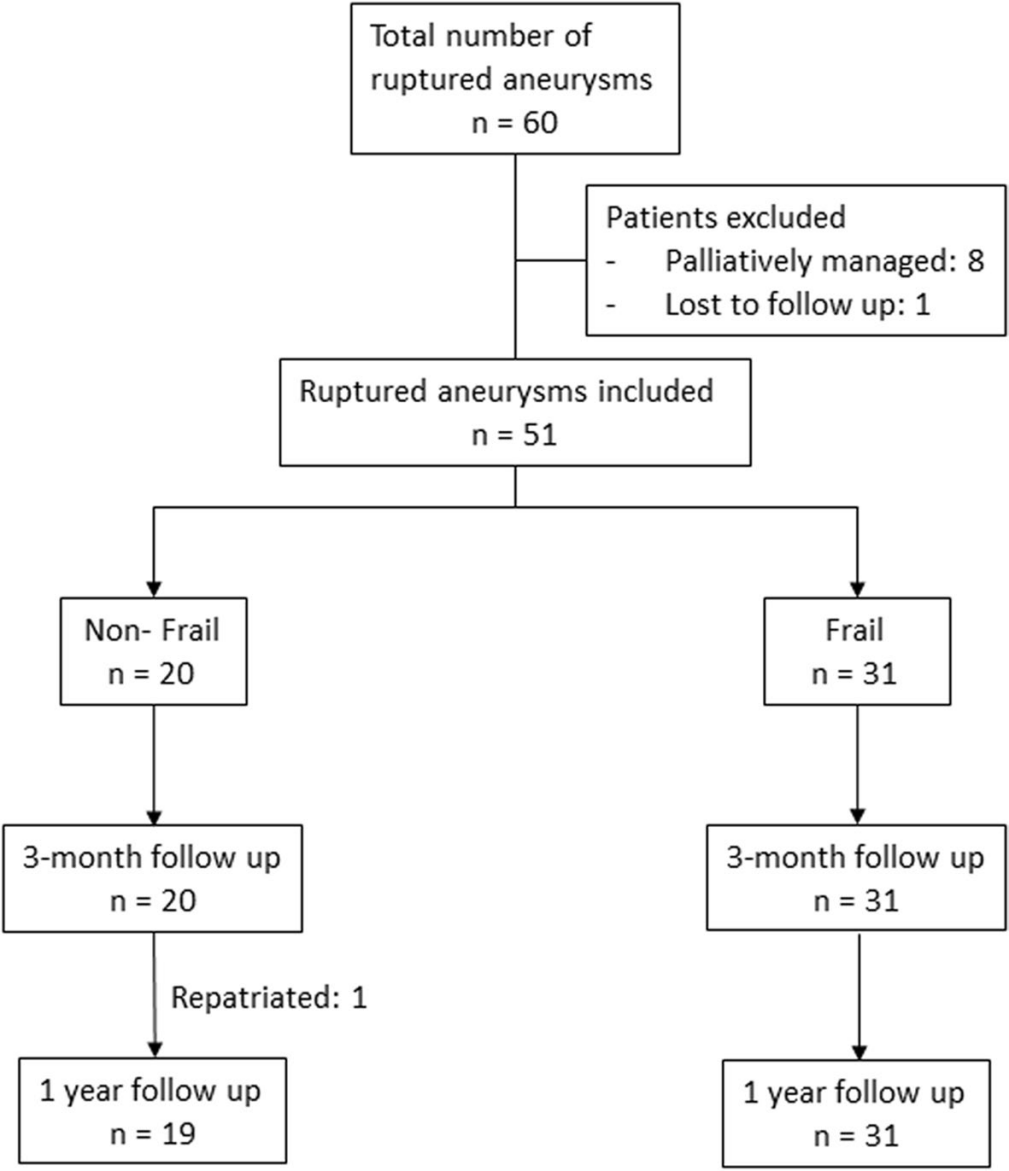
Patients and follow up at various timepoints

**Fig. 2:**
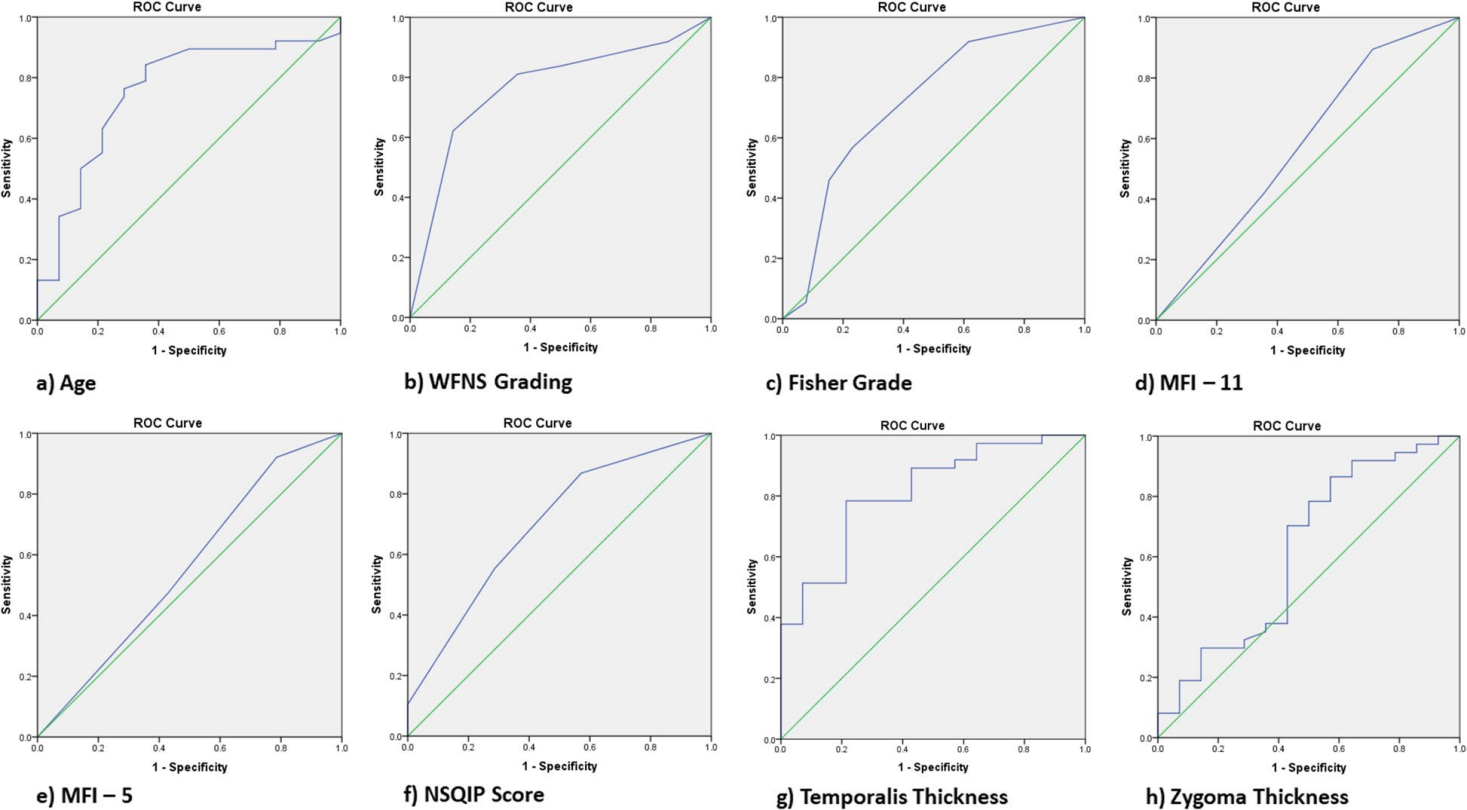
Receiver operating curve for **a**) age; **b**) WFNS; **c**) Fisher grade; d) MFI-11; **e**) MFI-5; f) NSQIP score; **g**) temporalis thickness; **h**) zygoma thickness. Area under curve (95% CI) of age: 0.76 (0.60 – 0.91); WFNS grade: 0.76 (0.62 – 0.91); Fisher grade: 0.72 (0.54 – 0.89); MFI-11: 0.58 (0.40 – 0.77); MFI-5: 0.56 (0.37 – 0.74); NSQIP score: 0.70 (0.54 – 0.86); TMT: 0.82 (0.70 – 0.94); ZGM: 0.62 (0.44 – 0.82) respectively. WFNS: World Federation of Neurological Surgeons: MFI: modified frailty index; NSQIP: National Surgical Quality Improvement Program; TMT: temporalis muscle thickness

There were 60 patients with ruptured intracranial aneurysms managed during this period. After applying the exclusion criteria, 51 patients were analysed (Fig. 1). When prediciting favourable mRS on discharge, the best performing marker in our cohort was TMT, with an AUC of 0.83 (0.70 – 0.94, *p* < 0.001), Somers’ D statistic of 0.63 and Tau’s statistic of 0.25, outperforming even traditional markers such as WFNS (AUC of 0.76, Somer’s D statistic of 0.53 and Tau’s statistics of 0.22). Amongst the frailty markers, NSQIP score performed the best, with an AUC of 0.69 (0.52 – 0.86, *p* = 0.039), Somers’ D statistic at 0.40 and Tau statistic at 0.16. This has a similar predictive value to another traditional aSAH prognosticators, modified Fisher scale (AUC of 0.68, Somers’ D statistic of 0.43 and Tau’s statistic of 0.17). MFI-11 and MFI-5, unexpectedly, did not perform well, with an unremarkable predictive value. Of note, when comparing the individual patient amongst the three frailty markers, the Spearman’s correlation demonstrated moderate to good agreement (MFI-11 vs. MFI-5, *r* = 0.91; MFI-11 vs. NSQIP, *r* = 0.69; MFI-5 vs. NSQIP, *r* = 0.75) (Table 4, Supp). The comparison of AUC results and ROC charts are further detailed in Table 3, Supp and Fig. 2, respectively.

Hence, TMT selected in the definition of frailty, with patients having a TMT < 5.5 mm deemed to be frail, and patients with TMT ≥ 5.5 mm deemed as non-frail. This threshold of 5.5 mm has a specificity of 0.93, sensitivity of 0.51, positive predictive value of 0.95 and negative predictive value of 0.42.

### Patient characteristics

There were 20 (39.2%) and 31 patients (60.8%) in the non-frail and frail groups, respectively. Both groups differed significantly in mean age (non-frail: 49.3 years, frail: 65.0 years; *p* < 0.001) and median WFNS grading on presentation (non-frail: 1 [1–2], frail: 2 [1–4]; *p* = 0.028). Otherwise, both groups were similar in terms of demographics, premorbid functional and medical status, and presenting clinical and radiological findings. Both groups also had a similar number of patients who underwent surgical clipping (Table 1).

**Table 1:** Characteristics of frail vs non-frail patients

		Overall	Non-Frail (*n* = 20)	Frail (*n* = 31)	OR (95% CI)	*P* Value
Age	Mean ± SD	58.8 ± 13.1	49.3 ± 11.1	65.0 ± 10.6	-	**< 0.001**
Male gender	Frequency (%)	10 (19.6)	6 (30)	4 (12.9)	2.89 (0.70 – 12)	0.16
Active smoking	Frequency (%)	4 (10.3)	1 (5.9)	3 (13.6)	0.40 (0.04 – 4.19)	0.62
mRS	Median (1Q – 3Q)	0 (0 – 0)	0 (0 – 0)	0 (0 – 0)	-	0.91
CCI	Median (1Q – 3Q)	0 (0 – 0)	0 (0 – 0)	0 (0 – 1)	-	0.20
Presenting GCS	Median (1Q – 3Q)	15 (9.25 – 15)	15 (14 – 15)	14 (9 – 15)	-	0.052
Anisocoria	Frequency (%)	4 (7.8)	2 (10.0)	2 (6.5)	1.61 (0.21 – 12)	0.64
WFNS grade	Median (1Q – 3Q)	2 (1 – 4)	1 (1 – 2)	2 (1 – 4)	-	**0.028**
Modified Fisher scale	Median (1Q – 3Q)	3 (1 – 3)	2.5 (1 – 3)	3 (1 – 3)	-	0.69
Hydrocephalus	Frequency (%)	16 (31.4)	6 (30.0)	10 (33.3)	0.86 (0.25 – 2.91)	0.80
Surgical clipping	Frequency (%)	26 (51.0)	13 (65.0)	13 (41.9)	2.57 (0.80 – 8.23)	0.11

*mRS* Modified Rankin scale, *CCI* Charlson comorbidity scale, MFI Modified frailty index, *GCS* Glasgow coma scale, *WFNS* World Federation of Neurological Surgeons

### Inpatient outcomes

After adjustment, the non-frail patients were found to be less likely to have delayed cerebral ischaemia (non-frail: 5.0%, frail: 32.3%, OR 0.11 [0.01 – 0.93]; *p* = 0.042) or any form of complications (non-frail: 30.0%, frail: 71.0%, OR 0.20 [0.06 – 0.69]; *p* = 0.011). Both groups were comparable in terms of the rate of neurocardiac syndrome, infection, the need for tracheostomy and ventriculoperitoneal shunt. Both groups also had similar NICU and overall length of stay and were equally likely to be discharged home or to a rehabilitation facility. The median mRS (non-frail: 1 [1 – 2.75], frail: 3 [2-4]; *p* = 0.011) and frequency of favourable mRS on discharge (non-frail: 95.0%, frail: 58.1%, OR 12.2 [1.39 – 107]; *p* = 0.024) favoured the non-frail group, while inpatient mortality was similar between groups (Table 2).

**Table 2:** Inpatient outcomes of non-frail vs frail patients

		Overall	Non- Frail (*n* = 20)	Frail (*n* = 31)	Unadjusted		Adjusted	
					OR (95% CI)	***P*Value**	OR (95% CI)	***P*Value**
Delayed cerebral ischaemia	Frequency (%)	11 (21.6)	1 (5.0)	10 (32.3)	0.11 (0.013 – 0.95)	**0.034**	0.11 (0.01 – 0.93)	**0.042**
Neurocardiac syndrome	Frequency (%)	4 (7.8)	1 (5.0)	3 (9.7)	0.49 (0.05 – 5.08)	1.00	0.80 (0.07 – 9.77)	0.86
Infective complications	Frequency (%)	21 (41.2)	6 (30.0)	15 (48.4)	0.46 (0.14 – 1.50)	0.19	0.50 (0.15 – 1.68)	0.26
Any overall complications	Frequency (%)	28 (54.9)	6 (30.0)	22 (71.0)	0.18 (0.05 – 0.60)	**0.004**	0.20 (0.06 – 0.69)	**0.011**
Tracheostomy	Frequency (%)	9 (17.6)	2 (10.0)	7 (24.1)	0.35 (0.06 – 1.89)	0.28	0.48 (0.08 – 3.07)	0.44
Ventriculopertioneal shunt	Frequency (%)	12 (23.5)	7 (35.0)	5 (17.2)	2.59 (0.68 – 9.79)	0.16	5.23 (0.97 – 28)	0.056
NICU length of stay	Median (1Q – 3Q)	8 (4.5 – 11)	8 (3 -11)	8 (5 – 14)	-	0.73	-	0.85
Overall length of stay	Median (1Q – 3Q)	24 (16 – 46.25)	18 (14.5 – 33)	28.5 (16 – 51.75)	-	0.24	-	0.53
Discharged home/ Rehabilitation	Frequency (%)	46 (90.2)	19 (95.0)	27 (93.1)	1.41 (0.12 – 16)	1.00	0.93 (0.07 – 12)	0.96
mRS on discharge	Median (1Q – 3Q)	2 (1 – 4)	1 (1 – 2.75)	3 (2 – 4)	-	**0.004**	-	**0.011**
Favourable mRS on discharge	Frequency (%)	37 (72.5)	19 (95.0)	18 (58.1)	13.7 (1.63 – 115)	**0.004**	12.2 (1.39 – 107)	**0.024**
Mortality	Frequency (%)	1 (2.0)	0 (0)	1 (3.2)	-	1.00	-	1.00

*NICU* Neuroscience intensive care unit, *mRS* Modified Rankin scale

### Long-term outcomes

**Fig. 3:**
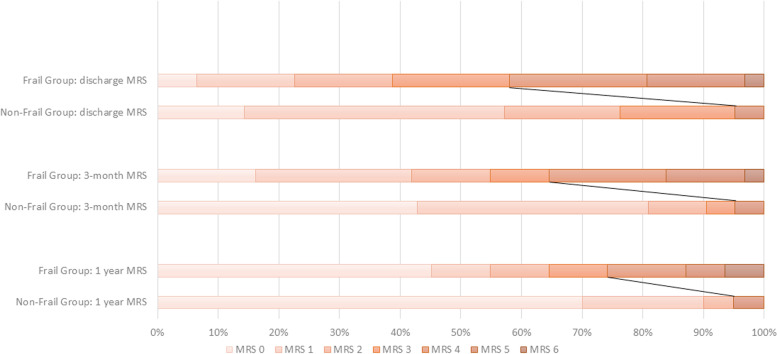
Comparison of modified Rankin scale for frail and non-frail groups at various timepoints. Oblique line demonstrates the threshold between favourable and unfavourable mRS (favourable mRS defined as 0 – 3). mRS: modified Rankin scale

Univariable analysis found both the median mRS and proportion of patients with favourable mRS at 3-month and 1-year to be associated with frailty. After multivariable analysis, only the median mRS at 3-months (non-frail: 1 [0 – 1], frail: 2 [1-4]; *p* = 0.015), and proportion of favourable mRS at 3-months (non-frail: 95.0%, frail: 64.5%, OR 9.01 [1.02 – 79]; *p* = 0.048) remained significant. However, this gap reduced with time; the difference between unfavourable and favourable MRS for frail vs. non-frail patients was insignificant at comparison of one year outcomes. This was reflected in the Grotta chart demonstrate in Fig. 3. Mortality at 30-day, 6-month, 1-year and 5-year intervals were similar amongst both groups (Table 3).

**Table 3:** Long-term outcomes in non-frail vs frail patients

						Unadjusted		Adjusted	
			Overall	Non-Frail (*n* = 20)	Frail (*n* = 31)	OR (95% CI)	*P* Value	OR (95% CI)	*P* Value
mRS	3 months	Median (1Q – 3Q)	1 (0 – 3)	1 (0 – 1)	2 (1 – 4)	-	**0.005**	-	**0.015**
	1 year	Median (1Q – 3Q)	0 (0 – 2)	0 (0 – 1)	1 (0 – 4)	-	**0.038**	-	0.064
Favourable mRS	3 months	Frequency (%)	39 (76.5)	19 (95.0)	20 (64.5)	10.5 (1.23 – 88)	**0.017**	9.01 (1.02 – 79)	**0.048**
	1 year	Frequency (%)	42 (82.4)	19 (95.0)	23 (74.2)	6.61 (0.76 – 57)	0.072	5.21 (0.54 – 50)	0.15
Mortality	30 day	Frequency (%)	1 (2.0)	0 (0)	1 (3.2)	-	1.00	-	1.00
	6 months	Frequency (%)	1 (2.0)	0 (0)	1 (3.2)	-	1.00	-	1.00
	1 year	Frequency (%)	2 (3.9)	0 (0)	2 (6.5)	-	0.52	-	1.00
	3 year	Frequency (%)	3 (5.9)	0 (0)	3 (9.7)	-	0.29	-	1.00
	5 year	Frequency (%)	5 (9.8)	0 (0)	5 (16.7)	-	0.14	-	1.00

*mRS* Modified Rankin scale

## Discussion

Multiple frailty indices and markers of sarcopaenia and osteopaenia have been investigated in the context of various surgical and neurosurgical disciplines. In this study, we utilized risk scores for frailty, and markers of sarcopaenia and osteopaenia, and compared them against traditional prognostication tools for outcomes following aSAH. We found that TMT was the best performing marker; it unexpectedly outperformed traditional aSAH markers, such as WFNS grading and correlated well with various inpatient outcomes. Of the frailty markers, NSQIP Score performed the best, at levels comparable to traditional aSAH markers with clinical relevance, such as Modified Fisher scale.

In the context of newer scales and novel measures gaining traction in clinical practice, such as frailty, traditional markers used to prognosticate the outcomes of aSAH, age, WFNS grade and Fisher scale remain relevant. This demonstrates the disproportionate role that the patients’ age and presenting status play in the overall outcomes from this condition. Unexpectedly, MFI-11 and MFI-5 were not well able to predict the outcomes in aSAH. This was also reported in a previous study of aSAH [24]. There are a few potential reasons that this might be the case. The MFI-11 has an over-representation of cardiac (history of congestive heart failure, myocardial infarction, previous percutaneous procedures, or angina) and neurological related variables (impaired sensorium, transient ischaemic attack or cerebrovascular accident, neurological deficit after previous cerebrovascular accident). Although both systems are critical in the overall patient outcomes, MFI-11 does not cover other important domains of frailty such as nutritional status, mobility, strength, and mood [10]. MFI-5 [12], derived from MFI-11, purported to have the same predictive power, suffers from the same shortcomings. In addition, both scores did not represent other important physiological systems such as the renal and hepatic systems. The NSQIP score had a better performance than both MFIs (AUC 0.70 [0.54 – 0.86] vs. 0.58 [0.40 – 0.77] and 0.56 [0.37 – 0.74]) possibly due to its incorporation of other factors that were able to indicate the patient’s non-neurological clinical status. These included variables that correspond to the patient’s nutritional status (recent weight loss and body mass index), other important physiological systems (renal, hepatic, haematological, etc.) and the acuteness of patient presentation (emergency case). The differences in performance between the NSQIP Score vs. MFI-11 and MFI-5 may well be attributable to the greater representation of systemic variables within the NSQIP Score, such as those correlated to susceptibility to infection (including systemic and wound infections, immunocompromised states such as disseminated cancer and steroid usage). Within the local context, frailty and mortality risks are known to be correlated with a high pathogenic load of latent infections [39].

Another reason that the MFI-11 and MFI-5 indices may fail to truly be indicative of the systemic load of comorbidities may be that these scores do not distinguish conditions by their severity. Patients with well-controlled hypertension, diabetes mellitus, congestive heart failure and chronic obstructive pulmonary disease were not distinguished in scoring from their poorly performing, chronically ill counterparts. Hence, MFI-11 and MFI-5 may not accurately reflect the supposed dynamic nature of frailty [40]. A further reason that frailty indices did not have the expected predictive value is the possibility that aSAH as an acute episode of brain injury overcomes the physiological reserves in a manner that having a frail or non-frail phenotype is inconsequential. This is likely true for patients who present with poor grade aSAH, where the major determinant for long-term good outcome remains the patients’ neurological status following response to resuscitation and amenability to interventions. This explains the findings of why frailty indices may correlate well with elective surgeries, which are clinical situations that do not stress the physiological reserves to the same extent as acutely ruptured intracranial aneurysms. Interestingly, other authors have demonstrated the relevance of frailty to higher frequency of presentation with poor grade aSAH [24]. In our study, grade of aSAH was indeed distinguished by frail vs. non-frail groupings. However, despite the differing presentations, as well as the increased burden of complications in the former group, there was a narrowing of the gap between them for longer-term outcomes. This, coupled with the relative importance of non-neurological factors in the NSQIP Score strongly suggest that, in our cohort, it may be possible to use such scoring towards implementing targeted interventions (such as to optimize nutrition and infection risks) to improve patient trajectories following aSAH.

Non-neurological factors such as nutritional status, may also explain why sarcopaenia, as measured here using TMT, demonstrated such a strong correlation with aSAH outcomes. The TMT marker supported the notion that non-frail patients were more likely to present with a favourable WFNS grade, less likely to experience delayed cerebral ischaemia and inpatient complications, were discharged at a better functional status and with faster recovery. Our results are consistent with published literature, in which sarcopaenia has been found to be reflective of clinical status and recovery in studies of patients undergoing rehabilitation [41, 42]. Deconditioning, which has been known to set in within the first day of admission [43] is demonstrative of the ability of sarcopaenia to reflect an acute to subacute context of frailty. In addition, our findings of sarcopaenia in this cohort are also consistent with local data demonstrating the correlation of measures of skeletal muscle mass with markers of subclinical vasculopathy, such as carotid intima-media thickness, albeit in an asymptomatic cohort [44]. Its capacity to encompass risk factors from multiple frailty domains, whilst also describing the dynamics of the physiological response to stressors, may therefore make sarcopaenia desirable as both a marker of muscle loss, as well as a surrogate for more global notions of an acutely “frail state”. Osteopenia, on the other hand, reacts much slower, possibly in the order of years. This is reflected in a study of the progression of osteopaenia in human immunodeficient virus infected patients using dual-energy X-ray absorptiometry [45]. Thus, it is not surprising that in acute non-traumatic brain injury, osteopaenia was not a good prognosticating factor for eventual clinical outcomes.

There were two unexpected observations in our study amongst both groups with regards to the inpatient and long-term outcomes. Firstly, despite frail patients having up to four times the rate of complications compared to the non-frail patients, the length of stay in the NICU and the ward, and the proportion of patients discharged home or to a rehabilitation facility were similar. We attribute this to the prompt and aggressive subspecialist clinical management of aSAH complications such as delayed cerebral ischaemia, neurocardiac syndrome and infections. Secondly, although frail patients had a significantly larger proportion with unfavourable mRS on discharge, after a year of rehabilitation and community interventions, the gap between the frail and non-frail patients narrowed. Furthermore, the proportion of non-frail patients with favourable mRS did not change from discharge up to the 1-year follow up. This suggests that frailty alone should not be threshold at which the offer of clinical interventions should be decided. We hypothesise that the reason for this phenomenon was that both groups had a similar proportion of patients with the rehabilitation potential, as evidenced by the similar proportion of patients with favourable mRS at the 1-year interval. However, it may be that non-frail patients were more able to reach this potential in a shorter time duration due to their enhanced physiological reserves at presentation. Whilst it may not be possible to augment the reserves of frail patients, our results suggest that it may be possible to develop specific interventions to buffer them through an acute state of worsening frailty and sarcopaenia, in order to reach their potential for long-term good outcomes.

Our study was limited by its retrospective nature and modest sample size. Although multivariable analysis was performed to reduce the effect of confounding, the modest sample size limited the ability to include more variables. While we explored the possibility of using other notable frailty indices such as risk analysis index, hospital frailty risk score, and the FRAIL questionnaire, data gaps in important domains such as poor appetite, cognitive deterioration, a general feeling of fatigue, and walking distance, limited our efforts to do so successfully. Nevertheless, our study sheds light on use of the concept of frailty in the context of acute brain injury from aSAH. There is a need for the understanding of how best to apply both global surrogates and specific markers indicative of the state of acute to subacute frailty and to understand their relevance to specific surgical contexts [46–48]. Furthermore, after confirmation with external validation, TMT has the potential to be included as a variable for a combined aSAH score that is able to predict peri-operative outcomes and hence assist in the risk stratification and management of patients with aSAH.

## Conclusion

Our study has demonstrated the immediate relevance for the utility of TMT as both a marker of outcomes following aSAH. Future work would include using other markers of sarcopaenia and examining patients with unruptured aneurysms to establish baseline thresholds for these factors as an important comparator cohort. As NSQIP score for frailty performs at similar levels to traditional prognostication tools for aSAH outcomes, it may be useful to further develop and refine its use within the context of a SAH-specific frailty index. Markers of sarcopaenia outperformed osteopaenia; TMT strongly correlated with the presenting status, inpatient, and long-term outcomes of aSAH, outperforming traditional tools. In this context of acute brain injury, TMT and NSQIP scoring may be valuable as as part of a risk stratification strategy to develop interventions promoting patient trajectories towards their best potential for good outcomes intervention.

## Supplementary Information

**Additional file 1. **[49].

## Data Availability

The datasets used and/or analysed during the current study are not publically available due to the active preparation of a follow up study but are available from the corresponding author on reasonable request.

## Abbreviations

CTA: Computed tomography angiography
MFI: Modified frailty index
mRS: Modified Rankin scale
NSQIP: National Surgical Quality Improvement Program
SAH: Subarachnoid haemorrhage
TMT: Temporalis thickness
WFNS: World Federation of Neurological Surgeons
ZGM: Zygoma thickness
