# Supplementary material for: Relevance of presenting risks of frailty, sarcopaenia and osteopaenia to outcomes from aneurysmal subarachnoid haemorrhage
Source: BMC Geriatr. 2022 Apr 16;22:333. doi: 10.1186/s12877-022-03005-7 (PMC9013113; doi:10.1186/s12877-022-03005-7)
Supplement: Supplementary file 1 — Additional file 1. [49]. [file 12877_2022_3005_MOESM1_ESM.docx]

**Supplementary Notes**

**Frailty indices and variables**

| MFI-11 [11] | MFI-5 [12] | NSQIP Score [13] |
| --- | --- | --- |
| ­History of congestive heart failure (1 point) | History of congestive heart failure (1 point) | History of congestive heart failure (2 points) |
| Hypertension requiring medications (1 point) | Hypertension requiring medications (1 point) | Anti-hypertensive medication use (1 point) |
| Non-independent functional status (1 point) | Non-independent functional status (1 point) | Non-independent functional status (1 point) |
| Diabetes mellitus (1 point) | Diabetes mellitus (1 point) | Ascites (7 points) |
| History of chronic obstructive pulmonary disease or pneumonia (1 point) | History of chronic obstructive pulmonary disease or pneumonia (1 point) | Ventilator dependence (4 points) |
| Myocardial infarction (1 point) |  | Renal failure (4 points) |
| Previous percutaneous procedure or angina (1 point) |  | Dialysis (3 points) |
| Peripheral vascular disease (1 point) |  | Bleeding disorder (2 points) |
| Impaired sensorium (1 point) |  | Emergency case (2 points) |
| Transient ischaemic attack or cerebrovascular accident (1 point) |  | Haematocrit <26% (2 points) |
| Neurological deficit after previous cerebrovascular accident (1 point) |  | SIRS/Sepsis/Septic shock (1 point) |
|  |  | Platelets <150,000 (1 point) |
|  |  | Wound infection (1 point) |
|  |  | Weight loss (1 point) |
|  |  | Body mass index <18.5 (1 point) |
|  |  | Age ≥65 years (1 point) |
|  |  | Disseminated cancer (1 point) |
|  |  | WBC ≥10,000 (1 point) |
|  |  | Steroid use (1 point) |

Table 1 (Supp): Variables involved in selected frailty indices

**Temporalis thickness measurement**

The temporalis muscle thickness (TMT) was measured using the axial cut of the admission computed tomography angiography of the circle of Willis (CTA) and the brain window was used. The TMT was then measured bilaterally, perpendicular to the long axis of the temporalis muscle, at the slice 5mm above the orbital roof, and the average of 3 measurements was taken [36]. Practically, the Sylvian fissure is an easily recognisable landmark adjacent to which the perpendicular line can be drawn. The mean (TMTmean), smaller and larger TMT (TMTsmall and TMTbig, respectively) were used in the AUC analysis. Please refer to Figure 1 (Supp)


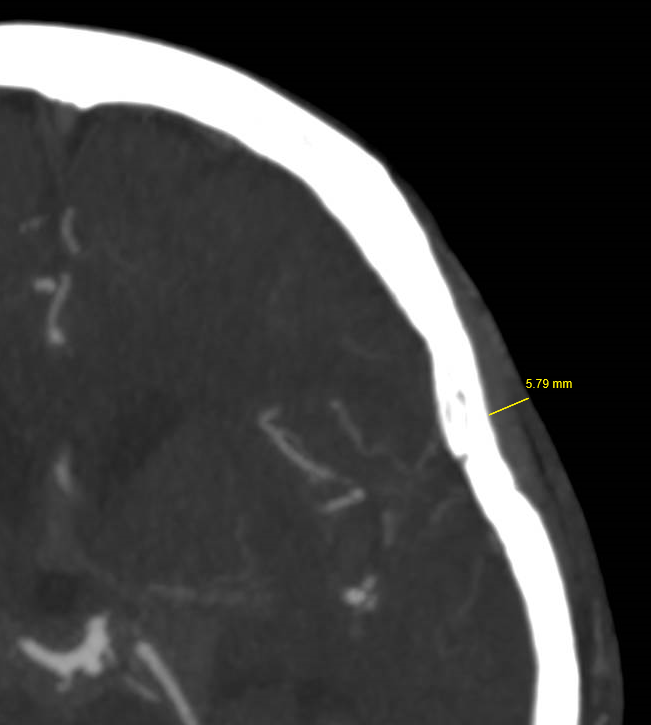


Figure 1 (Supp): Temporalis muscle thickness measurement

**Zygoma thickness measurement**

The zygoma thickness (ZGM) was measured also using the axial cut of the admission CTA. The bone window was used and the slice demonstrating the entire length of the thickest cut of zygoma was selected. ZGM width was taken at the midpoint and the mean (ZGMmean), smaller and larger ZGM (ZGMsmall and ZGMbig, respectively; regardless of laterality) were used in the AUC analysis. Please refer to Figure 2 (Supp).


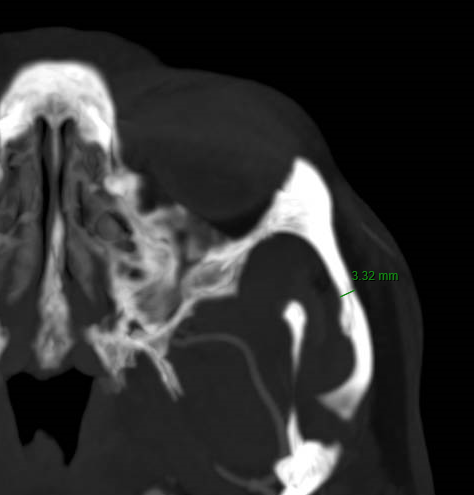


Figure 2 (Supp): Zygoma thickness measurement

**Inter- and intra-rater agreement measurement of temporalis and zygoma thickness**

Twenty patients were selected at random and their admission CTA was used in the performance of the inter- and intra-rater agreement analysis.

Interrater agreement was obtained from two study members independently (JXL and TMC) and intrarater agreement was obtained from a study member one week apart, with blinding to the initial result (JXL). The cut-off values for TMT and ZGM were obtained based on the AUC analysis as described in the statistical analysis. Cohen’s kappa was used, and values were interpreted as follows [49]: no agreement: κ ≤0; slight agreement: 0 < κ ≤0.20; fair agreement: 0.20 < κ ≤0.40; moderate agreement: 0.40 < κ ≤0.60; substantial agreement: 0.60 < κ ≤0.80; almost perfect agreement: 0.80 < κ ≤1.00. Both TMT and ZGM have demonstrated remarkable interrater and intrarater agreement over a range of values. Please refer to Table 2 for further details.

|  |  | Interrater Reliability | | Intrarater Reliability | |
| --- | --- | --- | --- | --- | --- |
| TMT | Cut-off (mm) | κ | P Value | κ | P Value |
|  | 3 | 1.00 | **<0.001** | 1.00 | **<0.001** |
|  | 4 | 1.00 | **<0.001** | 0.89 | **<0.001** |
|  | 5 | 0.89 | **<0.001** | 0.78 | **<0.001** |
|  | 6 | 0.86 | **<0.001** | 0.88 | **<0.001** |
| ZGM | 3 | 1.00 | **<0.001** | 1.00 | **<0.001** |
|  | 4 | 1.00 | **<0.001** | 1.00 | **<0.001** |
|  | 5 | 0.90 | **<0.001** | 0.90 | **<0.001** |
|  | 6 | 0.69 | **0.002** | 1.00 | **<0.001** |

Table 2 (Supp): Inter- and intra-rater agreement for TMT and ZGM

**AUC, Somers’ D and Tau analysis**

|  | AUC (SD) | Somers’ D Statistic | Tau Statistic |
| --- | --- | --- | --- |
| Age | **0.756 (0.072)** | **0.509** | **0.204** |
| WFNS | **0.762 (0.080)** | **0.533** | **0.216** |
| Modified Fisher scale | 0.684 (0.081) | 0.432 | 0.170 |
| MFI-11 | 0.592 (0.074) | 0.162 | 0.065 |
| MFI-5 | 0.578 (0.063) | 0.113 | 0.045 |
| NSQIP Score | 0.693 (0.077) | 0.395 | 0.158 |
| TMTmean | **0.818 (0.064)** | **0.625** | **0.254** |
| TMTsmall | **0.81 (0.071)** | **0.602** | **0.245** |
| TMTbig | **0.827 (0.059)** | **0.641** | **0.260** |
| ZGMmean | 0.646 (0.086) | 0.241 | 0.098 |
| ZGMsmall | 0.628 (0.077) | 0.185 | 0.075 |
| ZGMbig | 0.651 (0.094) | 0.255 | 0.104 |

Table 3 (Supp): Comparison of AUC analysis for SAH grading and various frailty indices, sarcopaenia and osteopaenia markers

Please refer to manuscript Figure 1 for the graphical representation of the AUCs for each individual predictor.

|  | Age | WFNS | Fisher | MFI-11 | MFI-5 | NSQIP Score | TMTbig | ZGMbig |
| --- | --- | --- | --- | --- | --- | --- | --- | --- |
| Age | 1.0 | 0.24  (p = 0.08) | 0.15  (p = 0.29) | 0.35  (p = 0.01) | 0.30  (p = 0.03) | 0.48  (p < 0.001) | - 0.51  (p <0.001) | - 0.24  (p = 0.09) |
| WFNS | 0.24  (p = 0.08) | 1.0 | 0.47  (p < 0.001) | - 0.17  (p = 0.23) | - 0.12  (p = 0.39) | 0.15  (p = 0.30) | - 0.34  (p = 0.01) | 0.02  (p = 0.88) |
| Fisher | 0.15  (p = 0.29) | 0.47  (p < 0.001) | 1.0 | - 0.20  (p = 0.17) | - 0.20  (p = 0.16) | 0.10  (p = 0.50) | - 0.07  (p = 0.63) | - 0.23  (p = 0.10) |
| MFI-11 | 0.35  (p = 0.01) | - 0.17  (p = 0.23) | - 0.20  (p = 0.17) | 1.0 | **0.91**  (p <0.001) | **0.69**  (p <0.001) | - 0.18  (p = 0.21) | - 0.00  (p = 1.00) |
| MFI-5 | 0.30  (p = 0.03) | - 0.12  (p = 0.39) | - 0.20  (p = 0.16) | **0.91**  (p <0.001) | 1.0 | **0.75**  (p <0.0001) | - 0.19  (p = 0.19) | 0.07  (p = 0.65) |
| NSQIP Score | 0.48  (p < 0.001) | 0.15  (p = 0.30) | 0.10  (p = 0.50) | **0.69**  (p <0.001) | **0.75**  (p <0.001) | 1.0 | - 0.41  (p = 0.003) | - 0.08  (p = 0.56) |
| TMTbig | - 0.51  (p <0.001) | - 0.34  (p = 0.01) | - 0.07  (p = 0.63) | - 0.18  (p = 0.21) | - 0.19  (p = 0.19) | - 0.41  (p = 0.003) | 1.0 | 0.09  (p = 0.54) |
| ZGMbig | - 0.24  (p = 0.09) | 0.02  (p = 0.88) | - 0.23  (p = 0.10) | - 0.00  (p = 1.00) | 0.07  (p = 0.65) | - 0.08  (p = 0.56) | 0.09  (p = 0.54) | 1.0 |

Table 4 (Supp): Agreement amongst various markers using Spearman’s correlation
